# Impact of synthetic surfactant CHF5633 with SP‐B and SP‐C analogues on lung function and inflammation in rabbit model of acute respiratory distress syndrome

**DOI:** 10.14814/phy2.14700

**Published:** 2021-01-06

**Authors:** Pavol Mikolka, Tore Curstedt, Riccardo Feinstein, Anders Larsson, Marian Grendar, Anna Rising, Jan Johansson

**Affiliations:** ^1^ Division for Neurogeriatrics Department of Neurobiology, Care Sciences and Society Karolinska Institutet Huddinge Sweden; ^2^ Biomedical Center Martin Jessenius Faculty of Medicine in Martin Comenius University in Bratislava Martin Slovakia; ^3^ Department of Physiology Jessenius Faculty of Medicine in Martin Comenius University in Bratislava Martin Slovakia; ^4^ Department of Molecular Medicine and Surgery Karolinska Institutet Karolinska University Hospital Stockholm Sweden; ^5^ Department of Pathology The Swedish National Veterinary Institute Uppsala Sweden; ^6^ Hedenstierna Laboratory Department of Surgical Sciences Uppsala University Uppsala Sweden; ^7^ Department of Anatomy, Physiology and Biochemistry Swedish University of Agricultural Sciences Uppsala Sweden

**Keywords:** ARDS model, CHF5633, inflammation, lung function, synthetic pulmonary surfactant

## Abstract

Acute respiratory distress syndrome (ARDS) is associated with diffuse inflammation, alveolar epithelial damage, and leakage of plasma proteins into the alveolar space, which together contribute to inactivation of pulmonary surfactant and respiratory failure. Exogenous surfactant delivery is therefore considered to hold potential for ARDS treatment, but clinical trials with natural derived surfactant or synthetic surfactant containing a surfactant protein C (SP‐C) analogue have been negative. Synthetic surfactant CHF5633, containing analogues of SP‐B and SP‐C, may be effective against ARDS. The aim here was to compare treatment effects of CHF5633 and animal‐derived surfactant poractant alfa in animal model of ARDS. ARDS was induced in adult New Zealand rabbits by mild lung lavages followed by injurious ventilation until respiratory failure (P/F ratio <26.7 kPa). The animals were then treated with intratracheal bolus of 200 mg/kg CHF5633 or poractant alfa (Curosurf^®^), or air as control. The animals were subsequently ventilated for an additional 4 hr and respiratory parameters were recorded regularly. Postmortem, histological analysis, degree of lung edema, and levels of the cytokines TNFα, IL‐6, and IL‐8 in lung homogenates were evaluated. Both surfactant preparations improved lung function, reduced the levels of pro‐inflammatory cytokines, and degree of lung edema to very similar degrees versus the controls. No significant differences in any of the analyzed parameters were observed between the CHF5633‐ and poractant alfa‐treated groups. This study indicates that single dose of CHF5633 improves lung function and attenuates inflammation as effectively as poractant alfa in experimental ARDS caused by injurious ventilation.

## 1 INTRODUCTION

Acute respiratory distress syndrome (ARDS) is a life‐threatening condition characterized by diffuse alveolar injury with bilateral pulmonary infiltrates on chest X‐ray and severe hypoxemia in the absence of evidence for cardiogenic pulmonary edema (Force et al., 2012). ARDS represents a stereotypic response to many different primary insults (e.g., pneumonia, gastric contents aspiration, sepsis, and hemorrhage) (Fanelli et al., 2013). The initial lung injury leads to deterioration of the alveolar‐capillary barrier by inducing dissolution of tight junctions as well as apoptosis and necrosis of alveolar epithelial type I and type II cells (Matthay & Zemans, 2011; Zemans et al., 2009) and development of alveolar edema, associated with loss of aerated lung tissue (Fanelli et al., 2013; Pierrakos et al., 2012). Migration and activation of neutrophils into the lung are critical components in ARDS and can result in degranulation and release of toxic mediators (e.g., proteases, reactive oxygen and nitrogen species, pro‐inflammatory cytokines, and pro‐coagulant molecules). This results in pulmonary surfactant inactivation, ventilation‐perfusion mismatch, and diffuse inflammatory reaction, which reduce lung compliance, increase physiological dead space, give rise to hypoxemia, and further affect lung function in the early phase of ARDS (Pierrakos et al., 2012; Verbrugge et al., 1997).

Therapeutic approaches for ARDS patients are based on mechanical ventilation, fluid‐restrictive strategies, and prone positioning, to prevent iatrogenic lung injury (Yadav et al., 2017). Given the high mortality rate of patients with severe ARDS, >40% (Villar et al., 2014), additional therapeutic approaches are needed. Patients with ARDS show injury to the alveolar epithelial barrier with effects on surfactant composition and function (Davidson et al., 2006), including altered phospholipid and fatty acid profiles, decreased levels of surfactant‐specific proteins, and impaired surface tension‐lowering properties (Gunther et al., 2001). Exogenous pulmonary surfactant treatment appears to be an effective adjunctive therapy, by increasing pulmonary compliance and reducing the inflammatory and edematous states (Davidson et al., 2006). Several randomized clinical trials of exogenous surfactant therapy in adults with ARDS have been conducted, and while it may improve oxygenation, no effects on mortality, duration of intensive care, or requirement for mechanical ventilation have been observed (Dushianthan et al., 2012; Meng et al., 2012). Factors that may influence the therapeutic effects of exogenous surfactant in ARDS include surfactant phospholipid and protein composition, biophysical activity, susceptibility to inactivation, and dose. Comparative trials for treatment of neonatal RDS show superiority of natural derived surfactants over protein‐free synthetic surfactants, most likely due to the presence of surfactant proteins (SP), SP‐B, and SP‐C in the former (Ardell et al., 2015). Poractant alfa is a natural derived surfactant from minced porcine lungs containing 1% SP‐B and SP‐C and has greatly improved outcomes in RDS patients (Singh et al., 2015). CHF5633 is a fully synthetic surfactant preparation consisting of dipalmitoylphosphatidylcholine and palmitoyl‐oleoyl‐phosphatidylglycerol, and peptide analogues of human SP‐B and SP‐C (Johansson & Curstedt, 2019; Sweet et al., 2017). Administration of CHF5633 resulted in marked improvement in lung expansion with the same efficacy as poractant alfa (Ricci et al., 2017a), and improved lung and brain injury scores (Rey‐Santano et al., 2017) in experimental neonatal RDS in preterm rabbits and lambs, respectively. A multicenter, double‐blind, randomized, controlled clinical trial of CHF5633 showed similar efficacy and safety as poractant alfa in preterm neonates with moderate to severe RDS and raised no safety concerns, with a promising clinical efficacy profile (Ramanathan et al., 2020; Sweet et al., 2017). In addition, the structures of the SP‐B and SP‐C analogues have been modified to increase hydrophobicity and at the same time avoid the risk of inadvertent oxidation (Johansson & Curstedt, 2019; Sato & Ikegami, 2012). Based on these results, we hypothesized that CHF5633 would be effective also in the treatment of ARDS. In addition, treatment of adult ARDS may require high amounts of exogenous surfactant, and natural derived surfactants are prepared by laborious extraction techniques (Dushianthan et al., 2012). Therefore, synthetic surfactants provide a potential alternative for providing large quantities of lung surfactant to a fair cost.

Herein, we tested this hypothesis by studying the treatment effects of CHF5633 and the animal‐derived surfactant poractant alfa on lung function and inflammation in a recently established experimental model of ARDS in adult rabbits (Zebialowicz Ahlstrom et al., 2019).

## 2 MATERIALS AND METHODS

### 2.1 Animals and Ethics

Twenty‐four adult New Zealand white rabbits, body weight (b.w.) 2.7 ± 0.2 kg, were used and handled according to the Federation of European Laboratory Animal Science Associations (FELASA) guidelines and recommendations (Guillen, 2012). All animal experiments were performed according to the ethical permit C76/16 obtained from the regional animal research committee (Uppsala Djurförsöksetiska Nämnd).

### 2.2 Surfactant preparations

Modified porcine surfactant poractant alfa (Curosurf^®^) and the synthetic CHF5633 surfactant containing SP‐B and SP‐C analogues (0.2 and 1.5% (w/w), respectively) mixed with 98.3% (w/w) dipalmitoylphosphatidylcholine and palmitoyl‐oleoyl‐phosphatidylglycerol in 1:1 mass ratio were used for treatment (Sato & Ikegami, 2012). Both surfactants were provided by Chiesi Farmaceutici.

### 2.3 General experimental setup

The animals were instrumented as previously described (Zebialowicz Ahlstrom et al., 2019). After initial anesthesia, tracheotomy was performed and left and right marginal ear veins and left ear artery were cannulated for continuous intravenous (i.v.) infusion of anesthetics (2 mL/kg/h, ketamine/xylazine), Ringer's acetate solution (10 mL/kg/h), blood sampling, and arterial pressure monitoring. The animals were mechanically ventilated (Servoi, Maquet Critical Care, Sweden) in volume‐controlled mode with a tidal volume (*V*_T_) of 10 mL/kg, positive end‐expiratory pressure (PEEP) of 5 cm H_2_O, respiratory rate (RR) of 30 breaths per minute (bpm), inspiratory to expiratory ratio (I:E) of 1:2, and inspired oxygen fraction (FiO_2_) of 0.7 for a 30 min stabilizing period. After that, FiO_2_ was increased to 1.0, and experimental ARDS was induced (see further below). After the treatment procedure, the animals were ventilated for additional 4 hr in volume‐controlled mode with a *V*_T_ 8 mL/kg, PEEP 5 cm H_2_O, RR 25–30 bpm, I:E 1:2, and FiO_2_ 1.0. PEEP was increased gradually up to 10 cm H_2_O in cases where oxygen saturation (SatO_2_) fell below 87%. Respiratory parameters and blood gases were recorded before (basal value, BV), after reaching ARDS, and every 30 min after administration of surfactant. Finally, the animals were euthanized under deep anesthesia by injection of potassium chloride *i*.*v*. All 24 animals survived the entire experimental procedure.

Monitoring of bio‐signals included electrocardiography, invasive arterial pressure, pulse oximetry, rectal temperature, and capnography (NICO; Philips Respironics). Gas exchange and parameters of acid–base balance were measured from arterial blood samples using blood gas analysis (Radiometer ABL 505; Radiometer OSM3). The following parameters were calculated: P/F as the ratio between arterial oxygen partial pressure (PaO_2_) and FiO_2_; dynamic lung‐thorax compliance (*C*_dyn_) as *V*_T_ /(peak inspiratory pressure (PIP)–PEEP); alveolar–arterial gradient (Nilsson et al., 1998) as [FiO_2_ (P_atm_–PH_2_O)–PaCO_2_/0.8]–PaO_2_, where *P*_atm_ is barometric pressure and PH_2_O is pressure of water vapor, and oxygenation index (OI) as (mean airway pressure × FiO_2_)/PaO_2_.

### 2.4 Experimental model of ARDS

Experimental ARDS was induced by a two‐hit model: repetitive mild lung lavages resulting in minimal surfactant depletion followed by high‐tidal volume injurious ventilation (Zebialowicz Ahlstrom et al., 2019). The lavages were performed with warm saline (5 mL/kg, 37°C) followed by suction and the process was repeated in total four times with stabilization periods in between (duration depending on SatO_2_), until P/F ratio decreased to <70 kPa. The animals were then ventilated with high‐tidal volume to mimic ventilator‐induced lung injury (VILI) in a pressure‐controlled mode with target *V*_T_ 20 mL/kg, PEEP 0 cm H_2_O, RR 20–30 bpm, I:E 1:2, and FiO_2_ 1.0. Hypocapnia was accepted without additional reduction of RR. Arterial blood gases were analyzed every 30 min until P/F in arterial blood decreased to <26.7 kPa.

### 2.5 Treatment procedure

Once the criteria of lung injury were fulfilled (P/F < 26.7 kPa), the animals were assigned randomly to one of the following three groups (n = 8 in each): (a) no surfactant treatment, air bolus (control group); (b) treatment with the natural modified surfactant poractant alfa alone; and (c) treatment with CHF5633 surfactant alone. Before and after surfactant administration, or air bolus in the control group, a lung recruitment maneuver was performed, 6 breaths at PEEP 10 cm H_2_O, PIP 30 cm H_2_O, RR 25 bpm, and I:E 1:2. Surfactant treatment (2.5 mL/kg, 200 mg phospholipids/kg) was given as bolus instillations in the trachea above the carina with the animal in semi‐upright right and in left lateral position (50% of the dose was given in each position). In the control group, an air bolus (2.5 mL/kg) was given instead of surfactant. After the treatment procedure, the animals were placed in prone position throughout the experiment and ventilated for 4 hr in volume‐controlled mode (*V*_T_ 8 mL/kg, PEEP 5 cm H_2_O, RR 25–30 bpm, I:E 1:2, and FiO_2_ 1.0).

### 2.6 Postmortem tissue sampling and assays

Anesthetized animals were euthanized by intravenous injection of a saturated potassium chloride solution. Immediately after disconnecting the ventilator and spontaneous lung collapse, the trachea was clamped above the carina level, and the lungs and heart were excised. Tissue samples from apical, medial, and caudal areas of right and left lungs were either immediately shock‐frozen in liquid nitrogen and stored at −70°C until biochemical analyses were performed, or immersed in 10% buffered formalin for 2 weeks for tissue fixation, or used to assess the degree of lung edema (see below).

Levels of cytokines were determined in a 10% (weight/volume) lung homogenate in 0.1 M phosphate‐buffered saline (PBS, pH 7.4). The concentrations of TNFα, IL‐6, and IL‐8 were quantified using rabbit‐specific ELISA kits (Cloud‐Clone Corp.) and expressed in pg/mL. The ELISA analyses were performed in duplicates and according to the manufacturers’ instructions.

Formalin‐fixed lung samples were embedded in paraffin, sectioned, and stained with hematoxylin and eosin. A quantitative morphometric analysis of interlobular and septal atelectasis/overdistension, and inflammation (based on leukocyte infiltration) was performed blindly by a veterinary pathologist (RF), and scored according to a five‐graded scale: 0 = not observed, + = mild, ++ = moderate, +++ = severe, and ++++ = very severe. The histological analysis of inflammation was done in a semiquantitative way based on features such as the number of inflammatory cells and the distribution and extension of cell infiltrates (i.e., a restricted area, scattered areas, patchy lesions, or diffusely distributed cell infiltrates). The total lung inflammation and atelectasis score were calculated as an average of the scores from the apical, medial, and caudal areas of the lungs.

Extent of lung edema was expressed as a wet‐to‐dry (W/D) lung weight ratio. Lung tissue samples from all harvested areas were weighed before and after drying in an oven at 42°C for 2 weeks to calculate the W/D ratio.

### 2.7 Statistical analysis

GraphPad Prism 6.01 (USA) and R ver. 3.5.2 with the aid of packages nlme and multcomp were used for statistical analysis. The results are presented as mean ±standard deviation (*SD*). Data normality was tested by Shapiro‐Wilk test. All assessed variables, except histological evaluation, were distributed normally in each group; therefore, we applied one‐way ANOVA with Welch's correction in order to test the differences between the groups and with Tukey post hoc test to test the differences between the groups in the parameters with dynamic changes for specific time points. Semiquantitative data from histological evaluation were tested by Kruskal‐Wallis test. Dependence of a variable on time was evaluated within the linear mixed model for each procedure, with the fixed effect of time, drug, and its interaction and the random effect allowing for uncorrelated shift and slope for each animal. Using Tukey test with Benjamini‐Hochberg correction of the *p* values, the same model was used to perform several comparisons of the mean of the variable between drug treatments. A *p* value below 0.05 was considered to be statistically significant. The effect size referring to the given relation has been quantified as eta‐squared with values from 0 to 1.0, indicating greater effects closer to 1.0. In Table S1, the specifics of statistical analysis including exact *p* values, confidence intervals (CI), and eta‐squared values (*η* ^2^) are presented. Trend estimates (for each treatment) and *p* values are shown in Table S2, using the linear mixed model with the fixed effect of time, drug, and their interaction and the random effect of the subjects. Estimates of the mean difference and corrected *p* values using multiple comparisons of the means of a variable between drug treatments using the linear mixed model are provided in Table S3.

## 3 RESULTS

### 3.1 Lung function parameters

**FIGURE 1:**
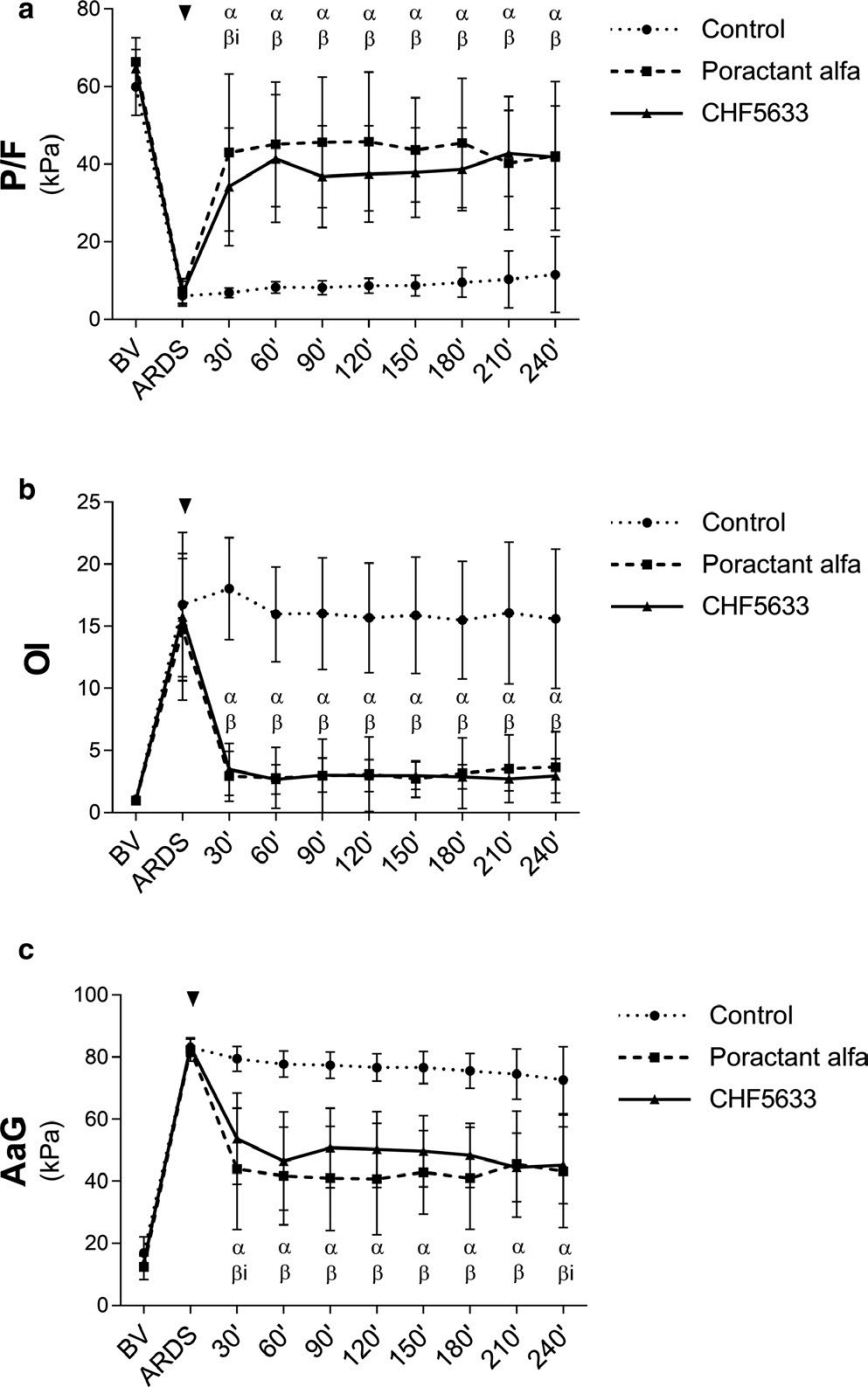
Respiratory parameters. (a) The ratio of arterial oxygen partial pressure to fraction of inspired oxygen (P/F, kPa), (b) oxygenation index (OI), (c) alveolar–arterial gradient (AaG, kPa) before (basal value, BV), at established ARDS, and during 4 hours after administration of surfactant therapy (marked with an arrow head). Data are presented as mean ±*SD*. Statistical comparisons: α for poractant alfa and β for CHF5633 represent *p* < 0.001 and βi for CHF5633 represent *p* < 0.01 vs. Control

In the initial phase of the experiments, there were no significant differences between the experimental groups in lung function parameters for the baseline values (BV) or at ARDS time point (control vs. poractant alfa vs. CHF5633, for all parameters *p* > 0.05). Severe deterioration in virtually all lung function parameters, including the ratio of arterial oxygen partial pressure to fraction of inspired oxygen (P/F), oxygenation index (OI), dynamic compliance (C_dyn_), alveolar–arterial gradient (Nilsson et al., 1998), airway pressure (Paw), and oxygen saturation (SaO_2_), was observed after induction of lung injury (ARDS time point vs. BV, *p* < 0.001). The P/F ratios after ARDS induction fulfill the criteria for severe ARDS according to the Berlin definition (Force et al., 2012). In the untreated group (control), the deterioration of all lung function parameters persisted for 4 hours till the end of the experiment (Figure 1, Table 1).

**TABLE 1:** Respiratory parameters monitored over time. Static lung‐thorax compliance (C_STAT_, ml/cmH_2_O), dynamic lung‐thorax compliance (C_DYN_, ml/cmH_2_O), mean airway pressure (Paw, kPa), partial pressure of oxygen (PaO_2_, kPa), carbon dioxide (PaCO_2_, kPa), oxygen saturation (SaO_2_, %), and arterial pH before (basal value, BV) and after induced ARDS and during 4 hrs after administration of therapy of untreated group (Control), and groups treated with poractant alfa or CHF5633 surfactant. Data are presented as means ±*SD*

	BV	ARDS	30′	60′	90′	120′	150′	180′	210′	240′
C_STAT_										
Control	2.8 ± 0.2	2.5 ± 0.1	1.9 ± 0.3	1.7 ± 0.1	1.7 ± 0.2	1.6 ± 0.1	1.5 ± 0.1	1.4 ± 0.1	1.4 ± 0.1	1.3 ± 0.1
Poractant alfa	2.7 ± 0.3	2.4 ± 0.1	2.0 ± 0.3	1.9 ± 0.3	1.9 ± 0.1*	1.7 ± 0.3	1.6 ± 0.3	1.5 ± 0.2	1.5 ± 0.2	1.4 ± 0.2
CHF5633	2.7 ± 0.2	2.4 ± 0.3	1.9 ± 0.2	1.8 ± 0.3	1.8 ± 0.2	1.7 ± 0.2	1.6 ± 0.2	1.5 ± 0.2	1.4 ± 0.2	1.4 ± 0.2
C_DYN_										
Control	2.8 ± 0.5	1.9 ± 0.2	1.5 ± 0.1	1.4 ± 0.1	1.4 ± 0.1	1.3 ± 0.1	1.3 ± 0.1	1.2 ± 0.2	1.2 ± 0.1	1.1 ± 0.1
Poractant alfa	2.6 ± 0.5	1.8 ± 0.1	1.7 ± 0.3	1.7 ± 0.3	1.7 ± 0.2	1.6 ± 0.3	1.5 ± 0.3	1.4 ± 0.2	1.4 ± 0.3	1.3 ± 0.3
CHF5633	2.9 ± 0.4	1.8 ± 0.2	1.8 ± 0.2	1.7 ± 0.3	1.6 ± 0.3	1.7 ± 0.3*	1.5 ± 0.3	1.5 ± 0.3	1.4 ± 0.3	1.3 ± 0.2
Paw										
Control	0.9 ± 0.0	1.2 ± 0.1	1.2 ± 0.1	1.3 ± 0.1	1.2 ± 0.1	1.3 ± 0.2	1.3 ± 0.2	1.3 ± 0.2	1.3 ± 0.2	1.3 ± 0.2
Poractant alfa	0.9 ± 0.1	1.2 ± 0.1	0.9 ± 0.1***	0.9 ± 0.1***	1.0 ± 0.1***	1.0 ± 0.1***	1.0 ± 0.1**	1.0 ± 0.1**	1.1 ± 0.1**	1.1 ± 0.1**
CHF5633	0.9 ± 0.0	1.2 ± 0.1	0.9 ± 0.1***	0.9 ± 0.1***	1.0 ± 0.1***	1.0 ± 0.1***	1.0 ± 0.1**	1.0 ± 0.1**	1.1 ± 0.1**	1.1 ± 0.1**
PaO_2_										
Control	41.9 ± 5.1	6.0 ± 1.9	6.8 ± 1.2	8.2 ± 1.4	8.2 ± 1.8	8.7 ± 1.9	8.7 ± 2.6	9.5 ± 3.8	10.3 ± 7.3	11.5 ± 9.8
Poractant alfa	46.4 ± 4.4	7.2 ± 3.3	43.0 ± 20.2***	45.1 ± 16.0***	45.6 ± 16.8***	45.8 ± 17.9***	43.7 ± 13.5***	45.4 ± 16.7***	40.3 ± 17.2***	42.1 ± 19.1***
CHF5633	45.2 ± 3.5	6.6 ± 3.2	34.1 ± 15.1**	41.4 ± 16.4***	36.8 ± 13.1***	37.5 ± 12.4***	37.8 ± 11.5***	38.7 ± 10.7***	42.7 ± 11.1***	41.7 ± 13.2***
PaCO_2_										
Control	5.8 ± 1.6	4.0 ± 0.9	6.1 ± 1.2	6.3 ± 0.9	6.6 ± 1.0	6.7 ± 1.1	6.7 ± 0.8	6.8 ± 0.9	7.0 ± 0.9	7.1 ± 0.7
Poractant alfa	6.0 ± 1.2	4.4 ± 1.4	6.7 ± 1.6	6.7 ± 1.7	6.9 ± 1.5	6.9 ± 1.2	6.8 ± 1.0	7.0 ± 1.2	7.1 ± 1.1	7.3 ± 1.3
CHF5633	6.4 ± 1.6	4.5 ± 1.0	6.3 ± 0.7	6.3 ± 0.7	6.5 ± 0.8	6.5 ± 0.6	6.6 ± 0.6	7.1 ± 0.7	6.9 ± 0.8	7.2 ± 1.2
SaO_2_										
Control	97.2 ± 1.5	75.6 ± 13.4	77.8 ± 6.7	84.9 ± 4.1	83.0 ± 6.4	85.3 ± 4.6	84.1 ± 5.1	84.9 ± 5.6	83.0 ± 5.9	84.1 ± 5.8
Poractant alfa	96.9 ± 1.6	80.3 ± 9.5	96.1 ± 1.7***	96.2 ± 2.2***	96.1 ± 2.1***	96.0 ± 2.4***	96.4 ± 1.6***	96.2 ± 2.3***	96.0 ± 2.1***	95.7 ± 2.0***
CHF5633	96.1 ± 1.4	78.2 ± 7.1	95.3 ± 2.0***	95.8 ± 1.5***	95.7 ± 1.5***	95.7 ± 1.5***	95.8 ± 1.5***	95.7 ± 1.5***	95.8 ± 1.5***	95.6 ± 1.6***
pH										
Control	7.5 ± 0.1	7.6 ± 0.1	7.4 ± 0.1	7.4 ± 0.1	7.4 ± 0.1	7.4 ± 0.1	7.3 ± 0.1	7.3 ± 0.1	7.3 ± 0.1	7.3 ± 0.1
Poractant alfa	7.5 ± 0.1	7.6 ± 0.1	7.4 ± 0.1	7.4 ± 0.1	7.4 ± 0.1	7.4 ± 0.0	7.4 ± 0.0	7.3 ± 0.0	7.3 ± 0.1	7.3 ± 0.1
CHF5633	7.5 ± 0.1	7.6 ± 0.1	7.4 ± 0.1	7.4 ± 0.1	7.4 ± 0.1	7.4 ± 0.1	7.4 ± 0.1	7.4 ± 0.1	7.4 ± 0.1	7.4 ± 0.1

Statistical comparisons: for Poractant alfa and CHF5633 vs. control.

* *p* < 0.05,

** *p* < 0.01,

*** *p* < 0.001.

Lung function was significantly improved after the administration of either surfactant preparation (Figure 1, Table 1). Marked and significant improvement in P/F, OI, AaG, Paw, and SaO_2_ was observed at the first analysis point after therapy (30’) in both poractant alfa and CHF5633 groups compared to controls, and persisted till the end of the 4 hr observation period (for all parameters at each time point after therapy, *p* < 0.01). CHF5633, but not poractant alfa, significantly improved C_dyn_ at 2 hours after the administration relative to controls (*p* = 0.014; CI: 0.07, 0.63), but the difference between the groups treated with CHF5633 or poractant alfa was small (Figure 1c). In contrast, only poractant alfa improved C_stat_ at 1.5 hours versus controls (*p* = 0.016; CI: −0.62, −0.06) (Table 1). Both surfactant preparations significantly improved lung function (P/F, OI, C_dyn_, AaG, Paw, PaO_2_, and SaO_2_) relative to controls when comparing the means using the linear mixed model with the fixed time effect (Table S3). There was no statistically significant time trend present in CHF5633‐treated animals as regards several primary respiratory parameters (P/F, OI, C_dyn_, AaG, Paw, PaO_2_, and SaO_2_), which indicates the continuity of the treatment effect. However, time trend analysis in poractant alfa‐treated group showed significant changes compared to controls (Table S2). When comparing the effects over time of the two surfactant preparations within the respective treatment group, no significant differences were observed for any lung function parameter and time points (for all parameters and time points *p* > 0.05).

### 3.2 Inflammatory cytokines

**FIGURE 2:**
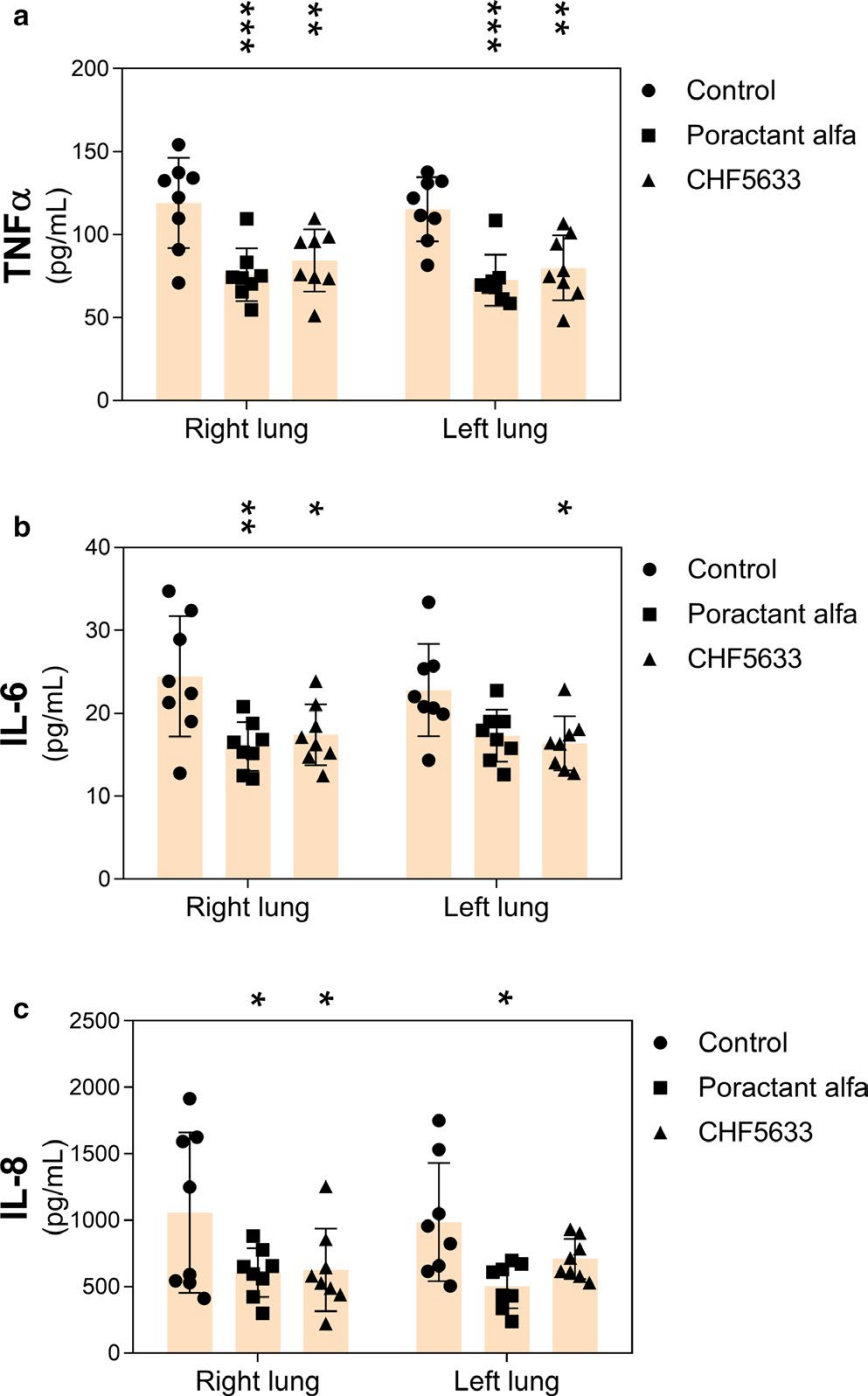
Inflammatory markers. Concentrations of cytokines (a) TNFα, (b) IL‐6, and (c) IL‐8 (all in pg/mL) in right and left lung tissue homogenates of untreated control group, and groups treated with poractant alfa or CHF5633 surfactant. Data are presented as individual values with mean ±*SD*. Statistical comparisons: for poractant alfa and CHF5633 vs. control *p < 0.05, **p < 0.01, ***p < 0.001

Administration of either surfactant preparation resulted in reduced levels of TNFα in both the right and left lung compared to the untreated controls (Figure 2a), and reduced levels of IL‐6 and IL‐8 in the right lung (Figure 2b,c). In left lung, significant reduction was observed for IL‐6 after CHF5633 treatment and for IL‐8 after poractant alfa treatment (Figure 2b,c). When comparing the levels of cytokines in whole lungs (data not shown), decreased levels of cytokines were observed after either surfactant treatment, *p*‐values for poractant alfa: TNFα (<0.0001; CI: 25.89, 60.00), IL‐6 (=0.0064; CI: 1.93, 11.94), and IL‐8 (=0.0075; CI: 133.2, 863.3); and *p*‐values for CHF5633: TNFα (=0.0002; CI: 16.71, 53.22), IL‐6 (=0.0093; CI: 1.59, 11.75), and IL‐8 (=0.0509; CI: −1.36, 760.9) compared to control group. There were no statistically significant differences between the surfactant preparations (poractant alfa vs. CHF5633, *p* > 0.05).

### 3.3 Histological analysis

**FIGURE 3:**
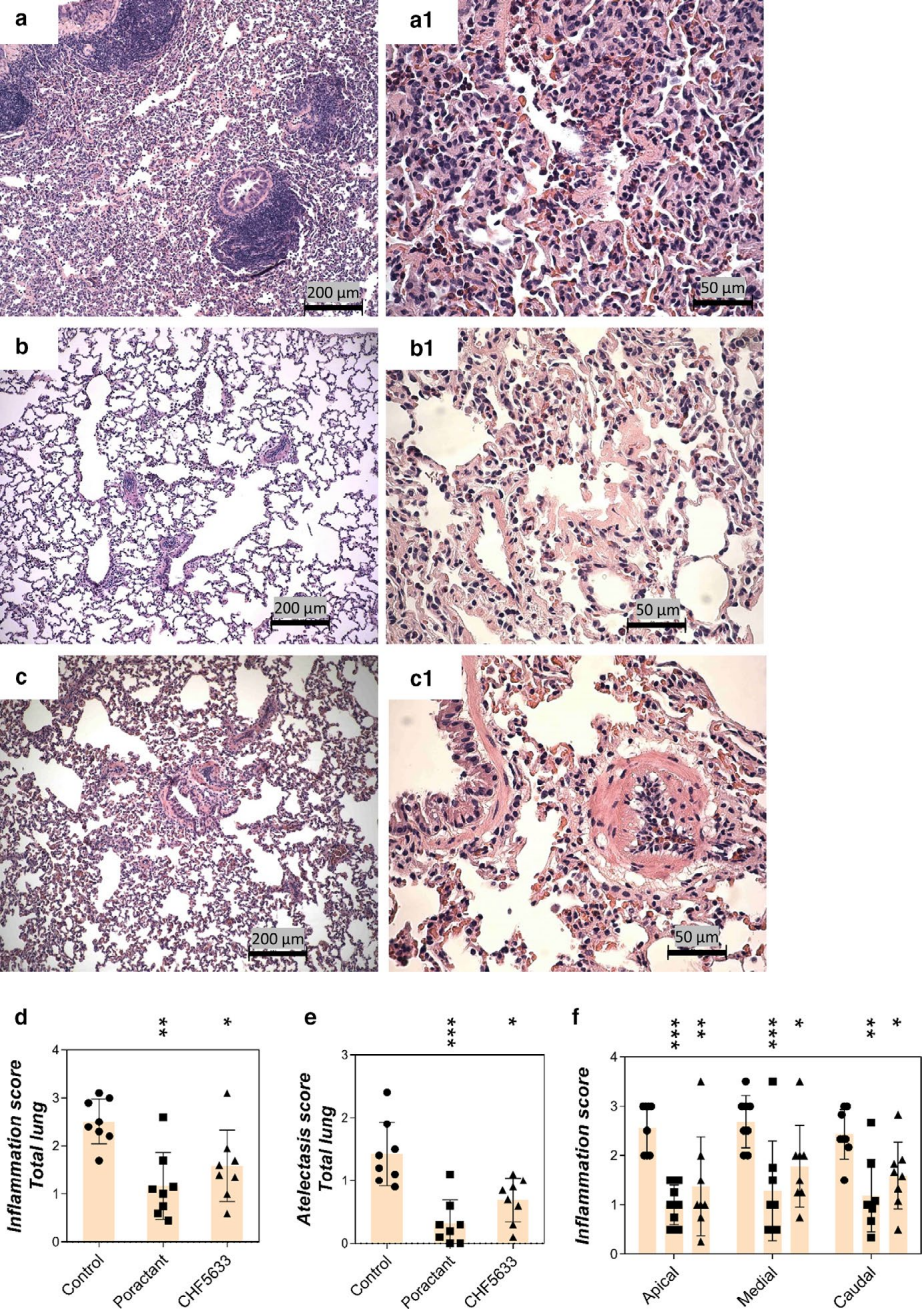
Histological analysis. Lung sections of untreated control group (a, a1), group treated with poractant alfa (b, b1), or CHF5633 surfactant (c, c1); total inflammation score (d), total atelectasis score (e), and inflammation score in apical, medial, and caudal regions of lungs (f). In control group, the pulmonary parenchyma displays a diffuse inflammatory cell infiltrate in alveoli and collapsed alveoli. Two bronchioles with prominent bronchial‐associated lymphoid tissue and small‐sized arteriole with a perivascular inflammatory cell infiltrate are observed (a). Alveoli with inflammation display numerous polymorphs, predominantly neutrophils and also some macrophages. Alveolar septa are markedly thickened with membranous deposits of eosinophilic proteinaceous material (a1). In the poractant alfa‐treated group, leukocytes are not visible at low power. The lung shows normal appearance, and consolidated areas are not observed (b). The central part shows membranous deposits of eosinophilic material in the alveolar septa. Scattered polymorphs also are visible (b1). In the CHF5633 treatment group, the pulmonary parenchyma shows normal alveoli with thin septa, and consolidated areas are not visible (c). The arteriole displays intravascular leukocytes, predominantly polymorphs, some of which adhere at the vascular endothelium. The pulmonary parenchyma is normal, with an occasional macrophage in the alveolar lumen (c1). The scale bars represent 200 μm in pictures a, b, and c and 50 μm in a1, b1, and c1. Data are presented as individual values with mean ±*SD*. Statistical comparisons: for poractant alfa and CHF5633 vs. control *p < 0.05, **p < 0.01, ***p < 0.001

Inflammatory cells were most prevalent in the alveoli, the interstitium around vessels (perivascular areas), and around the airways (peribronchial areas). Severe pneumonia was observed in the untreated control group and was characterized by neutrophils, eosinophils, and macrophages infiltrated in the pulmonary parenchyma (Figure 3a), consistent with an acute inflammation. Inflamed lungs displayed areas having thick alveolar septa. Thickened septa exhibited deposits of eosinophilic amorphous material. At sites, this material resembled hyaline membranes, but could also be precipitated blood proteins. Control rabbits showed numerous eosinophils in the cellular exudate (Figure 3a). Some rabbits had deposits of amorphous eosinophilic material in the alveolar lumina resembling fibrin. The pulmonary blood vessels showed prominent endothelial cells, in many cases associated with intense leukocyte infiltrates, consistent with vasculitis. These changes suggested that the vascular walls were involved in some form of reactive process and ongoing migration of leukocytes into the alveolar space.

Both surfactant preparations significantly attenuated the inflammatory processes (Figure 3b,c) in total (for poractant alfa *p* = 0.0017; CI: 5.55, 21.33; for CHF5633 *p* = 0.029; CI: 0.96, 17.54) and in apical, medial, and caudal regions of lungs compared to controls (Figure 3d,f). A significant reduction of atelectasis was observed after both surfactant therapies (for poractant alfa *p* = 0.0007; CI: 5.14, 16.86; for CHF5633 *p* = 0.0138; CI: 1.55, 13.08) (Figure 3e). Regional overdistension, caused by high‐volume ventilation, was reduced only after poractant alfa treatment versus controls (*p* = 0.0451; CI: 0.17, 8.08).

### 3.4 Lung edema formation

**FIGURE 4:**
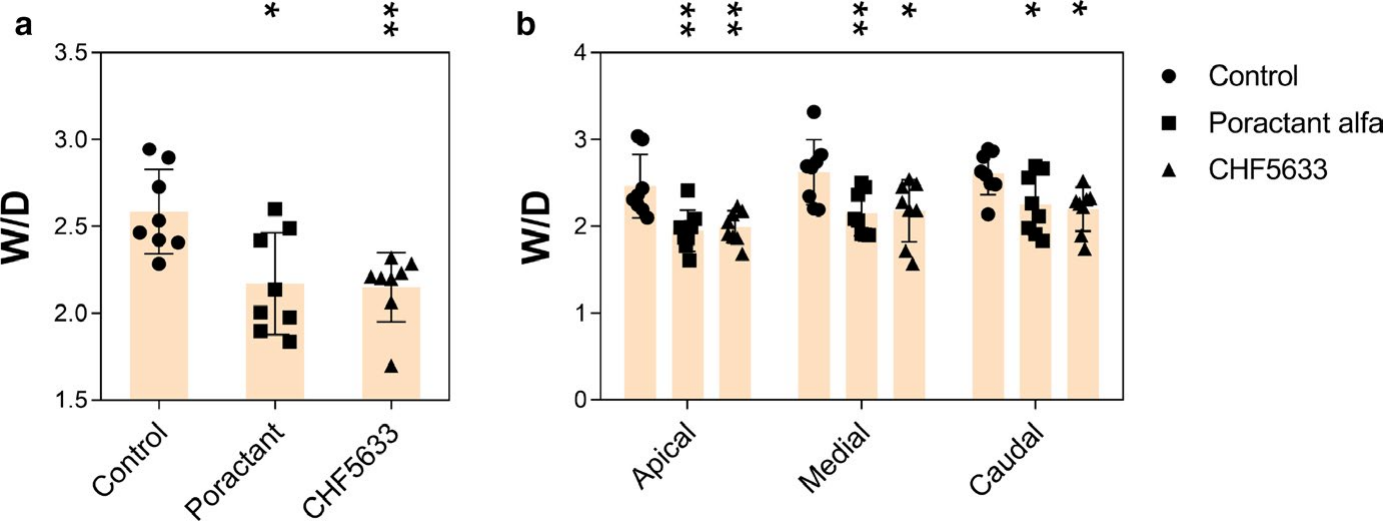
Lung edema formation. (a) Total lung edema expressed as wet‐dry (W/D) lung weight ratio, (b) W/D of apical, medial, and caudal regions of lungs of untreated control group, and groups treated with poractant alfa or CHF5633 surfactant. Data are presented as individual values with mean ±*SD*. Statistical comparisons: for poractant alfa and CHF5633 vs. control *p < 0.05, **p < 0.01, ***p < 0.001

Degree of lung edema was assessed by determining the wet‐dry lung weight ratio (W/D) of lung tissue from apical, medial, and caudal parts. Total lung edema formation was significantly reduced after both poractant alfa (*p* = 0.0216; CI: 0.06, 0.77) and CHF5633 surfactant (*p* = 0.0045; CI: 0.14, 0.72) treatments compared to the controls (Figure 4a), and the same effect was observed in all lung segments (Figure 4b).

## 4 DISCUSSION

ARDS is associated with diffuse epithelial damage caused by an insult to the alveolar‐capillary membrane, resulting in increased permeability and alveolar edema. Airspace infiltration by neutrophils amplifies and sustains the lung injury. In the acute exudative phase of ARDS, failure to repair the tissue damage results in ventilation‐perfusion mismatch and subsequent hypoxia (Fanelli et al., 2013; Villar et al., 2014). Self‐perpetuating inflammation, activated phospholipase A2, and plasma proteins leaking through the injured alveolar‐capillary membrane inactivate pulmonary surfactant and cause loss of lung function (Krafft, 2015; Seeds et al., 2012; Villar et al., 2016). Supportive therapies represent the mainstay of treatment of ARDS, the goal of which is to improve oxygenation and minimize iatrogenic injury, allowing effective treatment of the underlying cause of ARDS. Lack of effective ARDS therapies is likely linked to the complex pathogenesis of the syndrome (Confalonieri et al., 2017; Fanelli et al., 2013; Griffiths et al., 2019). Several randomized clinical trials of ARDS treatment with systemic glucocorticoids and exogenous surfactant have been conducted; however, conclusive support for efficacy has not been obtained (Dushianthan et al., 2012; Marik et al., 2008; Meng et al., 2012; Ruan et al., 2014). Using both natural and synthetic surfactant preparations, randomized clinical trials generally have shown improvements in oxygenation indices but have failed to produce any demonstrable survival benefits (Meng et al., 2012), even though the response to exogenous surfactant in direct lung injury preclinical studies has been promising (Bezerra et al., 2019; Kopincova et al., 2018; Mikolka et al., 2016; Zebialowicz Ahlstrom et al., 2019). Possible reasons for this failure of a potentially successful treatment include differences in surfactant composition, drug delivery methods, and variability in surfactant biology among the target population (Dushianthan et al., 2012).

In this study, we used a two‐hit experimental model of ARDS based on injurious ventilation (Zebialowicz Ahlstrom et al., 2019). The minimal lung lavage did not deplete pulmonary surfactant, but was intended to prime for the inflammatory lung injury induced by the following high‐volume lung ventilation leading to alveolar rupture, regional lung overdistension, plasma protein leakage, and inflammation (Slutsky & Ranieri, 2013), which could affect the function of the remaining endogenous surfactant and also the results of exogenous surfactant therapy. Thus, this model should be relevant for VILI and ventilated ARDS patients. After induction of ARDS, the animals were placed in prone position to increase homogeneity of ventilation and to improve gas exchange (Scholten et al., 2017). Despite this intervention, the lung function parameters of the control group, such as P/F, OI, AaG, and SaO_2_, remained deteriorated until the end of experiment (Figure 1), similarly to results obtained in previous studies (Kalk et al., 2008; Kamiyama et al., 2015; Ricci, Catozzi, et al., 2017; Zebialowicz Ahlstrom et al., 2019). Once the criteria of ARDS (P/F < 26.7 kPa) were fulfilled, surfactant therapy was administered as a bolus intratracheally and animals were ventilated for additional 4 hours. Treatment with poractant alfa and CHF5633 surfactants improved most lung function parameters markedly and to similar extents. Within the first 30 min after administration, we observed rapid improvement in P/F, OI, AaG, and SaO_2_ compared to control animals and this effect persisted until the end of experiment (Figure 1). Similar results were shown in previous studies of surfactant therapy in animal models of ARDS (Lutz et al., 1998; Nieman et al., 1996; Sun et al., 1996; Zebialowicz Ahlstrom et al., 2019). In a first‐in‐human clinical study of neonatal RDS, CHF5633 was efficacious, resulting in sustained improvements in oxygenation that occurred immediately after instillation. In terms of respiratory support, a shorter duration of non‐invasive ventilation was found in the 200 mg/kg CHF5633 cohort despite this group being slightly worse at baseline (Sweet et al., 2017). CHF5633 has also shown to be effective in treating extremely immature newborn lambs with surfactant deficiency (Sato & Ikegami, 2012). In the present study of experimental ARDS, the observed improvement in respiratory parameters indicates that animal‐derived poractant alfa and synthetic CHF5633 are similarly effective in improving lung mechanics (Figure 1, Table 1).

ARDS is associated with a significant local inflammatory reaction involving massive influx of leukocytes, especially granulocytes, which are homing to the place of injury and/or acute inflammation (Williams & Chambers, 2014). The histological analysis of the lungs 4 hr after induction of ARDS (Figure 3) supported this and we observed infiltrates of neutrophils, eosinophils, and macrophages in the pulmonary parenchyma, especially in the alveoli and the interstitium, particularly around blood vessels and bronchi. Introduction of a lung injury scoring system (Matute‐Bello et al., 2011) in future studies will allow further analyses of histological data. Activation of the infiltrated leukocytes is associated with increased production of pro‐inflammatory cytokines (Kalk et al., 2008; Kamiyama et al., 2015). Inflammatory processes contribute to diffuse alveolar damage and lung capillary endothelial injury, resulting in pulmonary edema formation (Cutts et al., 2017; Matthay & Zemans, 2011). Therefore, mitigation of inflammation in the acute phase of ARDS is desired and surfactant therapy with anti‐inflammatory action could be beneficial (Reid et al., 2005). In our study, both surfactant preparations decreased inflammation and atelectasis scores, reduced the level of pro‐inflammatory cytokines, and resulted in decreased lung edema formation (Figures 2, 3, 4). In line with this observation, previously published studies in vitro gave no evidence of any pro‐inflammatory effects, but rather indicated anti‐inflammatory features of CHF5633 on lipopolysaccharide (LPS)‐ or ureaplasma‐infected monocytes (Glaser et al., 2016, 2017). In this study, we focused on determining the level of inflammatory cytokines and edema formation as well as histological evaluation of lung tissue in different regions of lungs. We did not analyze bronchoalveolar lavage fluid for inflammatory cells, markers of biophysical activity, or resistance to inactivation, parameters that should be addressed in future studies. For further assessment of surfactant treatment of experimental ARDS, analysis of activity of phospholipase A2 could be beneficial for a link between inflammation and surfactant dysfunction (De Luca et al., 2013; Touqui & Arbibe, 1999).

Clinical studies have reported pulmonary benefits following the instillation of exogenous surfactants to children and adults with lung injury‐related acute respiratory failure or ARDS (Raghavendran et al., 2011). Randomized controlled trials of surfactant therapy in patients with ARDS have had limited success in improving long‐term outcomes, including survival, particularly in adults (Anzueto et al., 1996; Gregory et al., 1997; Kesecioglu et al., 2009). Due to the heterogeneous pathophysiology of ARDS, surfactants used for treatment must likely be able to resist inactivation by various compounds present in the alveoli in order to be effective. The resistance could depend on the presence of the hydrophobic SP‐B and SP‐C (or their analogues), which significantly enhance surfactant function in vitro, both independently and when combined (Schurch et al., 2010). Commonly used animal‐derived surfactant preparations contain both SP‐B and SP‐C (Walsh et al., 2013). The synthetic surfactant CHF5633 contains 1,2 dipalmitoyl‐glycero‐3‐phosphocholine (DPPC) and 1‐palmitoyl‐2‐oleoyl‐glycero‐3‐phospho‐1‐glycerol (POPG) (1:1), as well as 1.5% of an SP‐C analogue and 0.2% of an SP‐B analogue (Ricci, Murgia, et al., 2017). Use of synthetic surfactant may provide an alternative to animal‐derived product options. Moreover, CHF5633 has shown a higher resistance to albumin inactivation and a lower mortality rate in ventilated preterm lambs, compared to the natural surfactant poractant alfa (Seehase et al., 2012).

In conclusion, the aim of this study was to compare two surfactant preparations of different origins in terms of effects in an animal model of ARDS, focusing on lung function parameters, inflammation, and lung edema. The improvement in respiratory parameters and attenuation of inflammation were similar for animal‐derived poractant alfa and synthetic surfactant CHF5633, suggesting that CHF5633 and poractant alfa are similarly effective in this model of ARDS in adult rabbits. Using the same ARDS model, we recently showed that a synthetic surfactant that only contains an SP‐C analogue is somewhat less effective than poractant alfa (Zebialowicz Ahlstrom et al., 2019). However, in that previous study, two bolus doses were given instead of one as in the present study, which complicates further conclusions on relative efficacies of different surfactant preparations. Further studies using a more severe model of ARDS or a model that allows to monitor the animal for longer time or an infection model (bacterial or viral pneumonia) could be used to highlight the potential differences in therapeutic potential of different types of surfactant preparations.

## DISCLOSURE

Tore Curstedt and Jan Johansson are listed as inventors of patents held by Chiesi Farmaceutici on CHF5633. The authors alone are responsible for the content and writing of the paper.

## DATA FILES

Supplemental data include Tables S1, S2, and S3 that show the specifics of statistical analysis, trend estimates, and multiple comparisons of the means of a variable using the linear mixed model.

## AUTHOR CONTRIBUTIONS

AR and JJ conceived the study; PM performed the experiments; RF did histological evaluation; MG did statistical analysis; TC, AL, AR, and JJ supervised the work; PM did all artwork; PM, AR, and JJ wrote the manuscript, and all authors commented on the manuscript.
